# Dr. Charles Inslee Pardee

**Published:** 1899-12

**Authors:** 


Dr. Charles Inslee Pardee, one of the prominent physicians of New York, died suddenly at his home, No. 6 East 43d Street, November 4, 1899. He was apparently in his usual health attending to his professional duties up to the time he was taken suddenly sick, and he died in a half-hour after the attack.

Dr. Pardee was graduated from the New York University Medical School in 1860. He was a member of the Academy of Medicine and the Medical Society of the County of New York. He was also a member of the Military Order of the Loyal Legion, the New England Society and the Union League and Lotos Clubs. He was for many years dean of the New York University Medical School which some time ago was combined with the Bellevue Hospital Medical College. Dr. Pardee leaves a widow.

